# Searching for Hydrodynamic Orienting Effects in the Association of Tri-*N*-acetylglucosamine with Hen Egg-White Lysozyme

**DOI:** 10.1021/acs.jpcb.1c06762

**Published:** 2021-09-21

**Authors:** Beata Wielgus-Kutrowska, Urszula Marcisz, Jan M. Antosiewicz

**Affiliations:** Biophysics Division, Institute of Experimental Physics, Faculty of Physics, University of Warsaw, Pasteura 5 St., 02-093 Warsaw, Poland

## Abstract

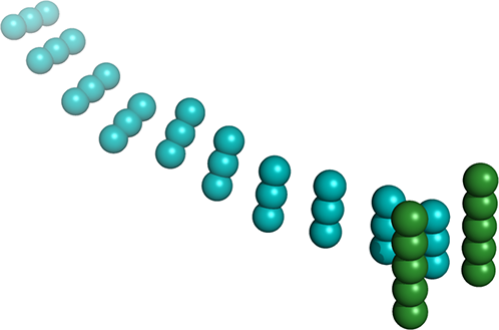

Using stopped-flow fluorometry, we determined rate constants for the formation of diffusional encounter complexes of tri-*N*-acetylglucosamine (NAG_3_) with hen egg-white lysozyme (*k*_a_^WT^) and its double mutant Asp48Asn/Lys116Gln (*k*_a_^MT^). We defined binding anisotropy, κ ≡ (*k*_a_^WT^ – *k*_a_^MT^)/(*k*_a_^WT^ + *k*_a_^MT^), and determined its ionic strength dependence. Our goal was to check if this ionic strength dependence provides information about the orienting hydrodynamic effects in the ligand-binding process. We also computed ionic strength dependence of the binding anisotropy from Brownian dynamics simulations using simple models of the lysozyme–NAG_3_ system. The results of our experiments indicate that in the case of lysozyme and NAG_3_ such hydrodynamic orienting effects are rather negligible. On the other hand, the results of our Brownian dynamics simulations prove that there exist molecular systems for which such orienting effects are substantial. However, the ionic strength dependence of the rate constants for the wild-type and modified systems do not exhibit any qualitative features that would allow us to conclude the presence of hydrodynamic orienting effects from stopped-flow experiments alone. Nevertheless, the results of our simulations suggest the presence of hydrodynamic orienting effects in the receptor–ligand association when the anisotropy of binding depends on the solvent viscosity.

## Introduction

**Figure 1:**
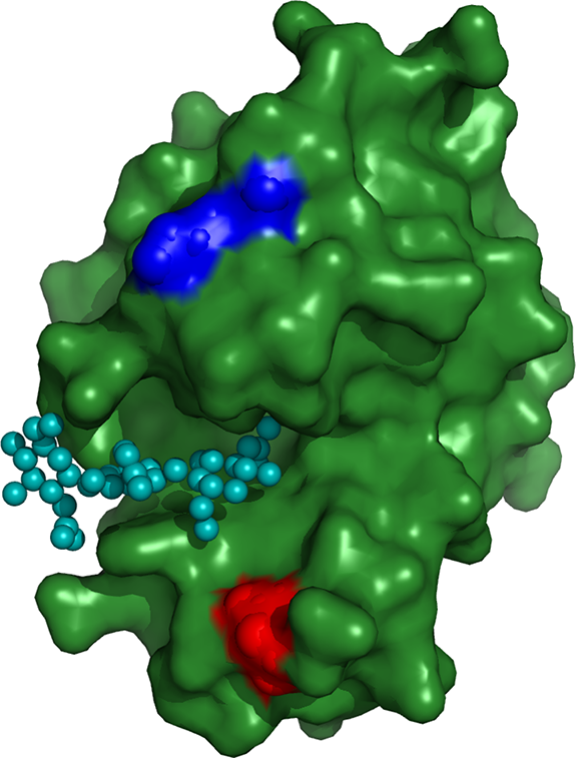
Deep elongated binding cleft of lysozyme molecule complexed with NAG_3_ ligand (cyan) and positions of the mutated residues marked with red (Asp48) and blue (Lys116). The image was prepared by using the 1HEW.pdb data file^7^ and the Pymol Molecular Graphic System, v0.99, Delano Scientific LLC.

The kinetics of binding of tri-*N*-acetylglucosamine (NAG_3_) to hen egg-white lysozyme (HEWL) was investigated previously by several groups.^1−5^ It is an interesting case of receptor–ligand association because for the reaction to occur the binding partners not only must be brought into close proximity but they also must assume a particular, sterically allowed relative orientation. The binding site of HEWL has a form of a deep cleft (see Figure 1) that runs across the entire width of the protein. The cleft is composed of six subsites, each capable of binding one *N*-acetylglucosamine residue.^6^

In general, for receptor–ligand association, a binding mechanism involving at least two steps is to be expected.^8,9^ The two-step binding is characterized by four rate constants: diffusional encounter rate constant, *k*_a_, for formation of an encounter complex, dissociation rate constant of the encounter complex, *k*_d_, conformational transition rate constant of the encounter complex, *k*_f_, and the reverse conformational transformation rate constant, *k*_b_, to the encounter complex, and, for HEWL and NAG_3_, can be represented by the following reaction equation:
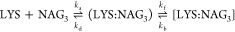
1In the present work, we are interested in the determination of the rate constants *k*_a_ for the HEWL–NAG_3_ systems, from our stopped-flow reaction progress curves, which will be treated as reliable estimations of the diffusional encounter rate constant. The diffusional encounter rate constant can be alternatively obtained from Brownian dynamics simulations.

Formation of the encounter complex is largely dictated by long-range electrostatic and hydrodynamic interactions. Electrostatic interactions result from the fact that each molecule can be treated as a distribution of electric charges. Hydrodynamic interactions result from the fact that each moving solute particle sets the solvent medium in motion, and the resulting flux in the solvent medium then tends to move all of the other solute particles. Depending on the net charges of associating molecules, electrostatic interactions can be either attractive of repulsive; thus, they can either speed up or slow down the association, whereas hydrodynamic interactions are generally expected to slow down the diffusional encounter.^10−12^ Additionally, for receptor–ligand pairs like that represented by HEWL and NAG_3_, the proper alignment of elongated ligand and the binding cleft, prior establishing specific short-range interactions in the final complex, can be achieved by orienting effects of electrostatic and hydrodynamic interactions. There is a vast literature documenting the role of electrostatic interactions, including the orienting effects, in the kinetics of molecular association.^13−26^ On the other hand, the existence or lack of existence of orienting effects of hydrodynamic interactions is not sufficiently documented yet.^27−32^

Here we explore a possibility to detect orienting effects of hydrodynamic interactions by a comparison of ionic strength dependence of the diffusional encounter rate constant for two related receptor–ligand systems. We investigate association of hen egg-white lysozyme and its double mutant Asp48Asn/Lys116Gln, with tri-*N*-acetylglucosamine in 20 mM glycine buffer, pH, 4.0, by a stopped-flow method with tryptophyl fluorescence observation of the transients. Simultaneously, we analyze two simple bead models of receptor–ligand with Brownian dynamics simulations employing the UHBD program,^33,34^ with two different variants of their electrostatic properties and the same hydrodynamic properties.

The idea of such investigation originates from the Brownian dynamics simulation study coauthored by one of the present authors.^29^ In that study, two receptor–ligand systems with the same electric charge distribution and differing with respect to their hydrodynamic properties were considered. The approach being checked now resulted from the understanding that it is rather impossible to influence and control the hydrodynamic torque between real molecules while keeping remaining hydrodynamic and electrostatic features intact.^35^ On the other hand, the influence and control of electrostatic torque effects in real experiments with associating molecules, while keeping their hydrodynamic features intact, e.g., by mutation of ionizable residues in proteins into related neutral amino acids, do seem feasible.

Figure 1 presents positions of Asp48 and Lys 116, which were mutated to Asn and Gln, respectively. The total net charges of HEWL and its double Asp48Asn/Lys116Gln mutant are expected to be the same. However, both proteins are expected to differ regarding the magnitude and orientation of their electric dipole moment. Simultaneously, the secondary and tertiary structures of both proteins are expected to be the same, what can be confirmed by far- and near-UV circular dichroism spectra.^36^ Therefore, we may expect that hydrodynamic properties of the lysozyme–NAG_3_ ligand system are the same for both variants of the protein.

On the basis of a comparison of the ionic strength dependencies for computed diffusional encounter rate constants with and without hydrodynamic interactions effects in the simulations, we want to check if experimentally determined dependencies allow us to make conclusions regarding presence of hydrodynamic steering effects^27^ possibly acting to orient elongated approaching ligand along the elongated binding cleft of the receptor.

## Materials and Methods

### Preparation of Solutions

Hen egg-white lysozyme (CAS Number 12650-88-3) was purchased from Sigma-Aldrich. *N*,*N*′,*N*″-Triacetylchitotriose (CAS Number 38864-21-0) was purchased from Toronto Research Chemicals Inc. Glycin (Art.-Nr. 3908.2), KCl (Art.-Nr. 6781.1), and NaOH (Art.-Nr. P031.1) were purchased from Carl Roth GmbH + Co. KG. HCl (CAS Number 7647-01-0) was purchased from Chempur, Poland. All reagents were used as received. All solutions were prepared with Millipore water. Lysozyme and NAG_3_ were dissolved in 20 mM glycine–HCl (pH 4.0). The solution ionic strength was established by adding an appropriate amount of KCl. The buffer without KCl added has the ionic strength of 6.7 mM. Stock solutions of NAG_3_ were prepared by dissolving the appropriate weight of crystalline solid in the glycine buffer of given ionic strength adjusted with KCl. Concentrations required for stopped-flow mixing experiments were obtained by serial dilution of the stock solution in the same glycine buffer.

### Preparation of the Mutant HEWL

D48N/K116Q was expressed in *E. coli* by using a designed and commercially ordered plasmid from GeneCust, Custom Services for Research (https://www.genecust.com/en/). The pure sample of the lysozyme mutant was obtained by solubilization of the inclusion bodies in buffer with 8 M urea, purification in two steps, i.e., ion exchange and gel chromatography, and refolding of the purified protein. Because the maximal concentration of the mutant HEWL we were able to prepare for our stopped-flow experiments was about 4 μM, also for the wild-type protein the same concentration was used.

### Spectrophotometric Measurements

UV–vis absorption spectra were recorded by using the UV-2401-PC Shimadzu spectrometer. Circular dichroism (CD) spectra were collected by using the Chirascan Plus (Applied Photophysics) spectrophotometer. Simultaneously with the CD spectra, the UV absorbance spectra were measured. For the far-UV range (190–250 nm) either a 0.1 or 1 mm cell was used. For the near-UV range (250–340 nm) a 10 mm cell was used. CD spectra of lysozyme solutions or corresponding solvent were scanned with 0.5 s integration, 0.5 nm step resolution, and 1 nm bandwidth. Six scans were performed and averaged. Prior to spectra measurements, the CD baseline was registered with an empty cell holder and with 3 s integration. From each recorded spectrum of HEWL solution, the corresponding smoothed buffer spectrum was subtracted. Buffer spectra were smoothed by the Savitzky–Golay method (window size 11)^37^ using the Pro-Data Chirascan 4.1 (Applied Photophysics Ltd.) software. All spectroscopic measurements were performed at 20.0 °C.

### Kinetic Experiments

A SX20 stopped-flow system (Applied Photophysics) was employed for the kinetic measurements. Samples were excited with a light-emitting diode (295 nm wavelength). The emission was collected at 90° to the excitation beam; a 320 nm cutoff filter was used (Schott WG 320). The excitation pathway was 5 mm, and the emission pathway was 1 mm. A 1:1 mixing ratio was used. The voltage of the photomultiplier was set to 500 V. Stopped-flow experiments consisted of mixing solutions of ∼4 μM of lysozyme with solutions of NAG_3_ of concentrations 4, 8, 16, 32, and 64 μM. The exact concentration of both variants of lysozyme in each experiment was established by absorbance measurements, assuming the molar extinction coefficient ϵ_280 nm_ = 37700 M^–1^ cm^–1^.^38^ For each pair of protein–ligand concentrations used in the stopped-flow experiments, 20 or more reaction progress curves were averaged. The resulting set of averaged progress curves represents one separate experiment. For each ionic strength and each lysozyme variant four such experiments were done. All solutions were prepared in degassed buffer. Each series of stopped-flow experiments was done by using freshly prepared protein samples. All measurements were performed at controlled temperature of 20.0 °C. The (averaged) reaction progress curves were analyzed numerically by using the DynaFit program^39,40^ and assuming a two-step binding model represented by eq 1.

### Electrostatic Calculations

#### HEWL

Atomistic models of hen egg-white lysozyme and its mutant were created based on the X-ray structure with PDB ID 1HEW(7) and by using molecular dynamics program CHARMM.^41^ To create the mutant structure, we changed the names of the appropriate amino acids and atoms in the pdf file and removed unnecessary atoms. Executing CHARMM, we added and optimized coordinates of lacking atoms in Asn48 and Gln116.

The electrostatic properties of proteins are determined to a large extent by the ability of certain amino acids to exchange protons with their environment and the dependence of these processes on pH. The charge distribution in lysozyme and its mutant corresponding to the experimental conditions was calculated by our computer methodology for titration of proteins, as described in full detail elsewhere.^42,43^ The required electrostatic calculations were performed by using the finite-difference Poisson–Boltzmann (PB) method,^44^ implemented in the University of Houston Brownian Dynamics program (UHBD).^33,34^ All simulations were performed at 293 K, with a solvent dielectric constant of 78, and that for the protein 2. The dielectric boundary between the protein and the solvent is defined as a Richards probe-accessible surface^45^ with a 1.4 Å probe radius and an initial set of 280 surface dots per atom.^46^ All atomic partial charges and radii for the protein were taken from the CHARMM27 parameter set for the standard amino acids and nucleic acids.^47,48^ The titration curves and the electric dipole moments, referred to the center of diffusion,^49^ of lysozyme and its mutant were computed as described elsewhere.^50^

#### NAG_3_

The atomistic model of the NAG_3_ molecule was created based on the same crystallographic structure as the HEWL model, 1HEW.^7^ Partial charges assigned to NAG_3_ atoms were taken from the work of Zhong et al.^51^ These charges were used to evaluate the net total charge and the permanent dipole moment of the NAG_3_ molecule.

### Models of the Receptor and Ligand

Simulations of diffusional encounters including hydrodynamic receptor–ligand interactions for realistic models of HEWL and NAG_3_ are not possible at present; therefore, we used a substantially simplified model. Simultaneously our model maximizes hydrodynamic orienting effects and thus serves as a useful reference system.

**Figure 2:**
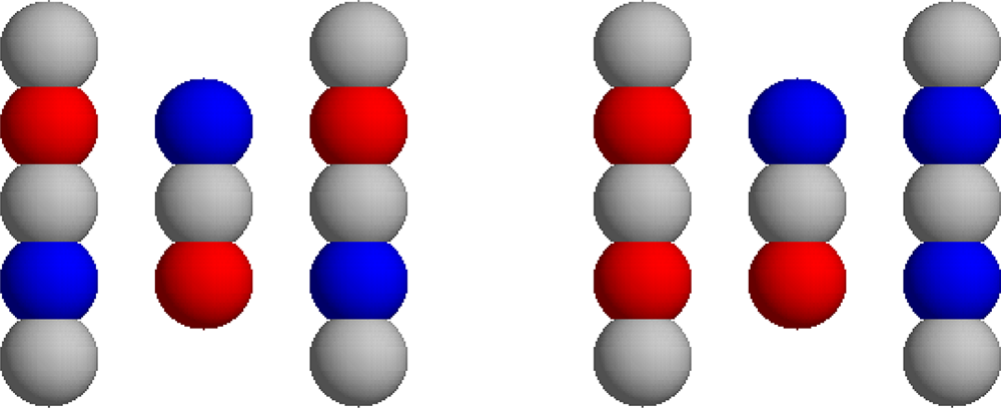
Bead models of elongated binding cleft and elongated ligand. Two electrostatic variants with dipole moments antiparallel (left) and perpendicular (right) for arrangement in the complex.

Hydrodynamic models of the receptor with elongated binding cleft and its elongated ligand employed in Brownian dynamics simulations were built of overlapping spherical elements of equal radii of 2 Å. In the case of the ligand, this is a linear array of three beads, which corresponds to three subunits of tri-*N*-acetylglucosamine. The receptor is modeled by two linear arrays of five beads each, parallel to each other. These two arrays are separated by 12 Å. In each array, neighboring beads are separated by distance of 3 Å. Figure 2 presents two electrostatic versions of the receptor model with models of the ligand bound centrally between the two arrays of five beads. Our simple models are expected to maximize orienting hydrodynamic interactions between the receptor and the ligand approaching its binding site.^27,32^ Moreover, we expect that the electrostatic and hydrodynamic orienting interactions both promote orientation of the ligand shown in Figure 2 on the left. For the electrostatically changed model of the receptor, shown on the right, only hydrodynamic orienting interactions force the approaching ligand to keep parallel orientation with respect to the binding cleft.

The beads in Figure 2 are colored according to their assumed electric charges: red color means a negative charge of −2*e* located in the center of the corresponding bead, blue color means positive charge of +2*e*, and gray color means zero charge. In the complex arrangement shown on the left-hand side of Figure 2, the permanent electric dipole moments of the ligand and receptor are antiparallel, which corresponds to the minimum of their electrostatic interaction energy. On the right-hand side the dipole moment of the receptor cleft is rotated 90°counterclockwise. Therefore, in the mutual arrangement of the ligand and receptor shown on the right-hand side of Figure 2, the dipole moment of the receptor is perpendicular to the dipole moment of the ligand in the complex arrangement.

### Brownian Dynamics Simulations

Simulations of molecular diffusion and calculation of the rate constant for the diffusion-controlled encounter for bead models of the receptor and ligand are based on the Brownian dynamics (BD) method,^52,53^ implemented in the UHBD program,^34^ which was modified, as described earlier,^28,29^ to include hydrodynamic interactions between associating molecules. Both interacting molecules are represented by spherical bead models, as described above. Hydrodynamic interactions between beads used in modeling diffusing molecules were approximated as pairwise additive contributions described by Rotne–Prager tensors.^54^ We realize that usage of pairwise additive Rotne–Prager hydrodynamic tensors to model hydrodynamic interactions is a rather crude approximation. However, employment of more sophisticated description of the hydrodynamic interactions^55,56^ can hardly be used in Brownian dynamics simulations in three-dimensional Cartesian space^32,57^ because it would make the time necessary to complete the results unacceptably long. On the other hand, even our simplified Brownian dynamics approach is useful and enables reliable interpretation of experimental data.^58^

To obtain diffusional encounter rate constants from BD simulations with the UHBD program, one computes and then analyzes many trajectories of one reactant diffusing toward its partner “receptor” under the influence of electrostatic intermolecular forces and the random forces mimicking the influence of the bombardment by the solvent molecules. The computation of the diffusional encounter rate constants is based on the ratio of trajectories ended with the “reaction” to the total number of trajectories.^34^

We used three reaction criteria for our receptor–ligand model. The fulfillment of each successive reaction criterion means that the previous one was also fulfilled. Such an approach allows us to follow the history of the diffusion of the ligand near the binding site of the receptor.

**Figure 3:**
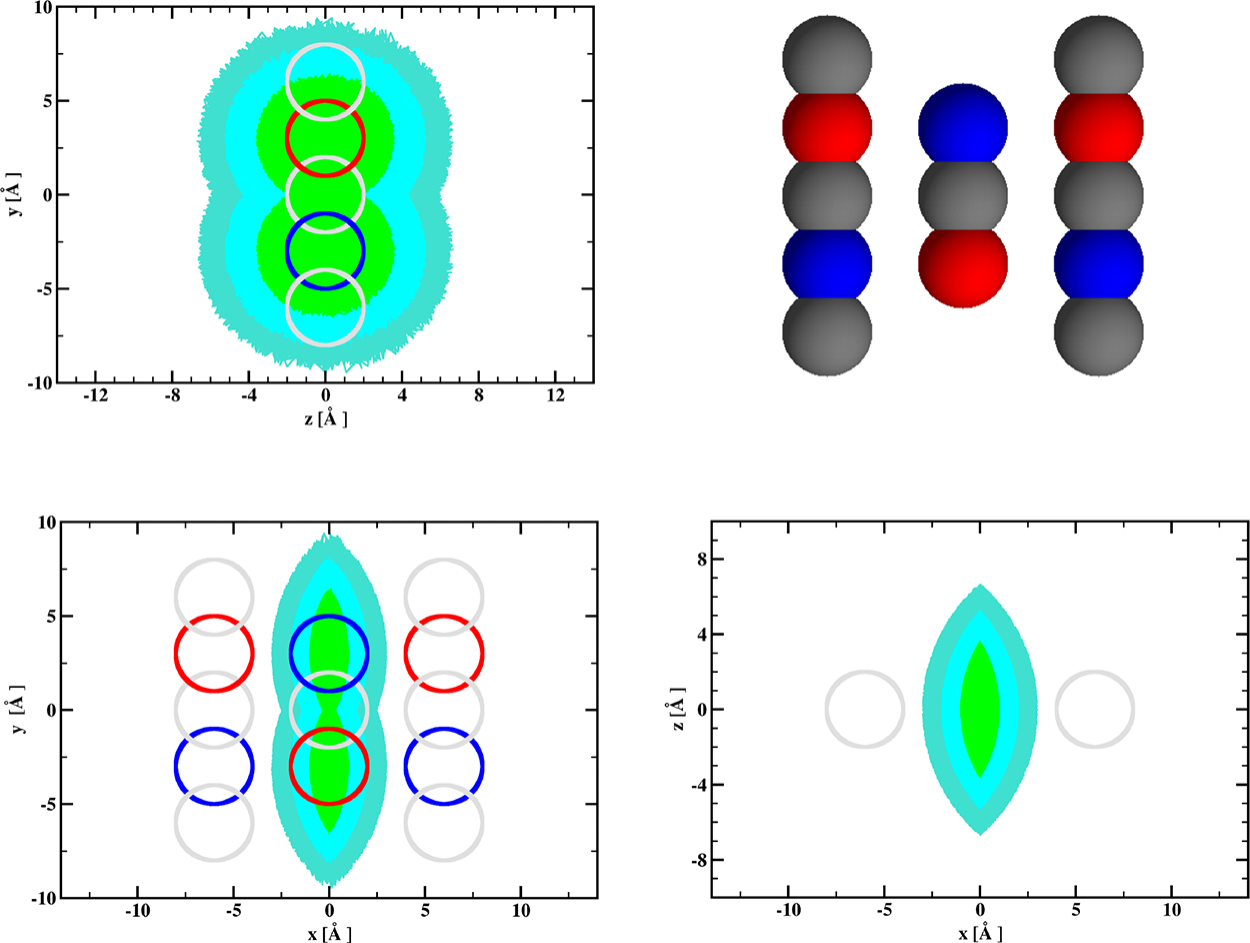
Diffusional encounter reaction criteria used in the present work (see text for details).

Figure 3 presents three perpendicular views of the three reaction criteria used in the simulations. The green color shows the most restrictive reaction criterion (No. 3). It shows positions of the centers of the two extreme beads of the ligand model when, simultaneously, its negatively (positively) charged bead is at the distance not exceeding 7 Å from any of the positively (negatively) charged beads of the receptor model. Subsequent, less restrictive reaction criteria set these limiting distances to 8 Å (cyan, No. 2) and 9 Å (turquoise, No. 1). The boundaries of the green, cyan, and turquoise areas are not sharp because they represent positions of 50000 pairs of randomly sampled points representing the two extreme beads of the ligand model, which satisfy the given reaction criterion. As can be seen, for the most restrictive reaction criterion, to satisfy the reaction criteria, the ligand must keep centers of its beads close to the *xz*-plane, but simultaneously the angle its long axis makes with the Cartesian Oy axis can be as large as 70°. We did not set more restrictive reaction criterion because it would result in substantially smaller number of reactions in our Brownian dynamics simulations and much worse statistics.

The reaction criteria used for the second electric model of the receptor are defined analogously, but it should be noted that charged beads of one array of the receptor change their charge on the opposite one, and consequently the dipole moment of the second model of receptor has its electric dipole moment rotated by 90° relative the first model.

As a reference, simulations that neglect the hydrodynamic interactions between receptor and ligand were also performed. Examples of inputs for both types of simulations are shown in the Supporting Information. Diffusional rate constants reported in the present work are based on 600000 Brownian trajectories. All simulations were performed at 293 K at a desired ionic strength. The solvent viscosity was set to 1.002 cP to represent water at 293 K.

## Results and Discussion

### Electric Properties of Wild-Type and Mutated Lysozyme and of NAG_3_

The total charge of the WT HEWL at pH 4.0 at 293 K changes from +9.0*e* for the ionic strength 0 through +10.9*e* for 150 mM and +11.3*e* for 500 mM. For the mutated HEWL these charges are +8.5*e*, +10.7*e*, and +11.1*e*, respectively. The values of the electric dipole moments are at these ionic strengths: 107.5, 154.1, and 168.6 D for the WT and 99.1, 93.0, and 99.5 D for the mutant. These dipole moments are computed relative to the diffusion center^49^ of the molecule. The angle between the dipole moment of the wild-type and that of the mutant is 45° for zero ionic strength, 35° for 150 mM ionic strength, and 31° for 500 mM ionic strength.

NAG_3_ has zero net charge; thus, its permanent dipole moment can be computed with position vectors of its partial atomic charges taken relative to an arbitrary origin of the Cartesian coordinate system. The calculated magnitude of the NAG_3_ dipole moment is 18.7 D, while the magnitude of the dipole moment of its first monomer is 5.03 D. The monomer result compares well with values given by Zhong et al.,^51^ who obtained values in the range 5.1–5.4 D for different conformations of the NAG monomer.

### Circular Dichroism Spectra of the Wild-Type Lysozyme and Its D48N/K116Q Double Mutant

**Figure 4:**
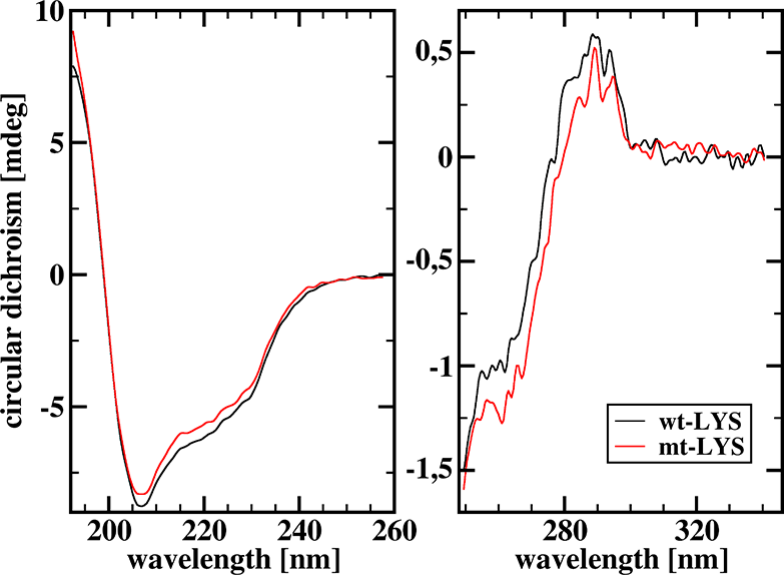
Comparison of the far- and near-UV circular dichroism spectra of 4 μM solutions of the wild-type hen egg-white lysozyme and its D48N/K116Q double mutant.

We registered far- and near-UV spectra (Figure 4) for the wild-type hen egg-white lysozyme and its mutant at concentrations of 4 μM, without addition of KCl. The CD spectra of the wild-type lysozyme registered in this study are essentially the same as previously reported.^59−62^ Moreover, the spectra of the wild-type lysozyme and its mutant, for both ranges, the far-UV CD and the near-UV, are very similar. That indicates that both the secondary and tertiary structures of lysozyme and its mutant are pretty much the same. Therefore, we conclude that hydrodynamically both variants of the protein might be considered identical; thus, for a given mutual distance of the ligand and lysozyme, and mutual orientation of the ligand and the elongated binding cleft, the hydrodynamic interactions between associating partners are very much the same.

**Figure 5:**
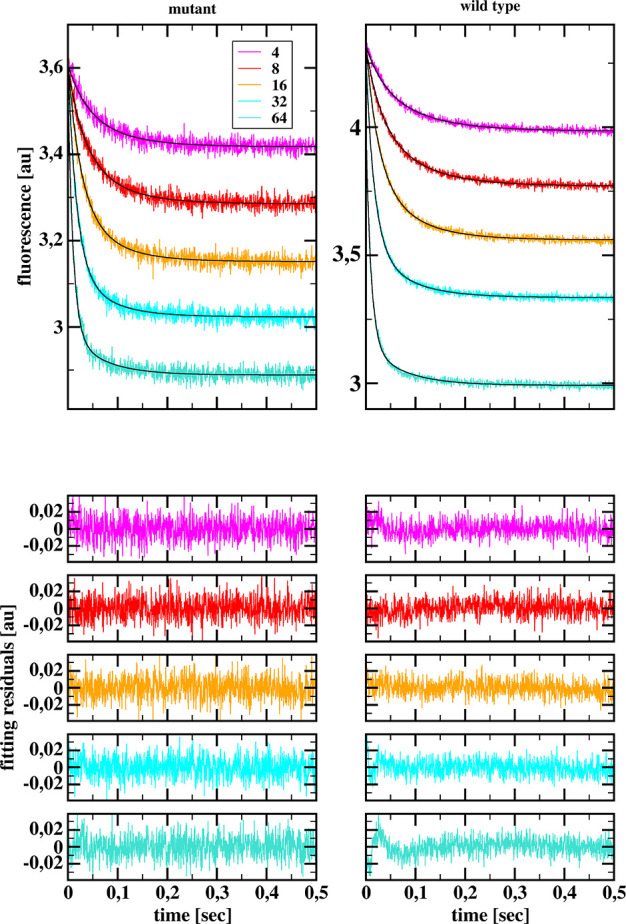
Example of the fluorescence progress curves obtained for the wild-type and mutated hen egg-white lysozyme (top) after mixing 4 μM protein solution with a solution of tri-*N*-acetylglucosamine, having concentration in μM indicated in the inset, together with two-step association model fits (black continuous lines) and residuals for the fits (bottom).

Probably relevant for our discussion of the identity or lack of identity of the secondary and tertiary structures of the hen egg-white lysozyme and its Asp48Asn/Lys116Gln double mutant is the observation that the fluorescence of the mutant is clearly smaller than the fluorescence of the wild-type lysozyme (see Figure 5).

### Results of Stopped-Flow Fluorometry

In Figure 5 we show an exemplary sets of HEWL–NAG_3_ reaction progress curves registered in stopped-flow experiments conducted at solution without addition of KCl (ionic strength = 6.7 mM). Each progress curve shown in this figure is the average progress curve obtained from at least 20 mixings in the stopped-flow fluorometer cell.

Analysis of these two particular sets of progress curves with the DynaFit^39,40^ program and the reaction model represented by eq 1 gives the diffusional encounter rate constant for formation of the encounter complex, 2.59 ± 0.04 and 2.77 ± 0.02 μM^–1^ s^–1^ for the mutant and wild-type, respectively. The result obtained for the wild-type lysozyme is close to the average value obtained in our previous study,^63^ 2.98 ± 0.08 μM^–1^ s^–1^, at pH 3.2 and 20 °C, in the glycine–HCl buffer without addition of KCl.

According to the model discrimination analysis implemented in the DynaFit program, in the case of the wild-type lysozyme, the three-step binding model, i.e., containing one more conformational transition step in comparison the two-step model of eq 1, is the most probable. For this three-step model, the estimation of the diffusional encounter rate constant gives 3.49 ± 0.08 μM^–1^ s^–1^. However, because our progress curves registered for the mutant lysozyme are of worse quality than those registered for the wild-type lysozyme, the DynaFit program never indicated for them the three-step model as superior and sometimes indicated the one-step binding model as the best one. Estimations of the diffusional encounter rate constants, when we compare the wild-type and the mutated lysozyme, should be derived from the same binding model. As a compromise, we analyzed all registered progress curves with the two-step binding model shown by eq 1.

Table 1 presents average diffusional encounter rate constants for the wild-type and mutated lysozyme with tri-*N*-acetylglucosamine, derived from four series of independent stopped-flow experiments, as functions of the ionic strength. It can be seen that for each ionic strength the rate constant for the wild-type lysozyme is larger than that for the mutated lysozyme, but except for *I* = 200 mM, the error ranges overlap. However, the dependence of both rate constants on the ionic strength is not very regular. It is most probably related to the relatively substantial standard error of our results. The results for the wild-type lysozyme agree very well with the results obtained in our previous work^63^ for slightly lower pH (3.2) and twice larger concentration of the protein.

**Table 1:** Diffusional Encounter Rate Constants and Their Standard Errors Obtained for Reactions of Tri-*N*-acetylglucosamine with the Wild-Type Hen Egg Lysozyme, *k*_a_^WT^, and Its Double Asp48Asn/Lys116Gln Mutant, *k*_a_^MT^ (See Text for Details)

ionic strength [mM]	*k*_a_^WT^ [μM^–1^ s^–1^]	std err [μM^–1^ s^–1^]	*k*_a_^MT^ [μM^–1^ s^–1^]	std err [μM^–1^ s^–1^]
6.7	2.96	0.17	2.44	0.31
10.0	2.86	0.16	2.76	0.19
20.0	2.77	0.13	2.68	0.11
50.0	2.84	0.12	2.56	0.31
75.0	2.73	0.12	2.71	0.12
100.0	2.79	0.17	2.58	0.13
150.0	2.96	0.04	2.77	0.32
200.0	2.95	0.04	2.71	0.04
300.0	3.00	0.12	2.93	0.12
500.0	3.12	0.16	3.04	0.17

The results presented in Table 1 were converted to the binding anisotropy, defined by eq 2:
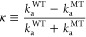
2

**Figure 6:**
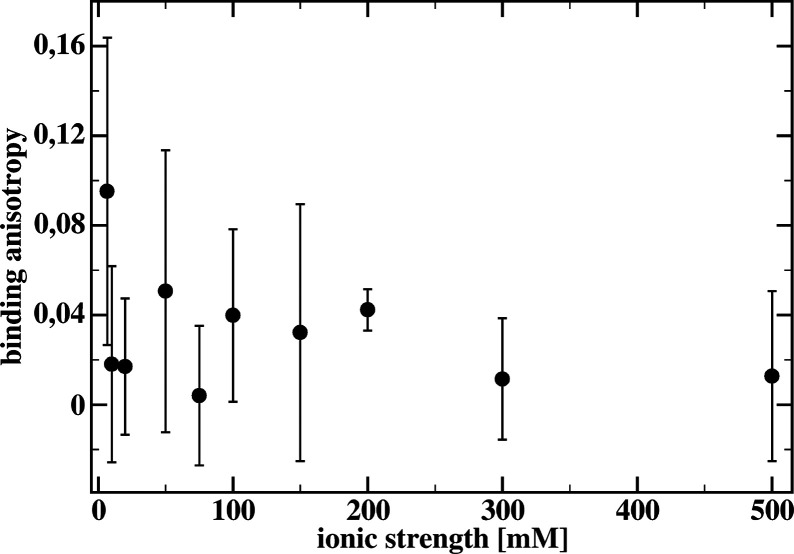
Binding anisotropy κ defined by eq 2 as a function of the ionic strength. See text for details.

The dependence of the binding anisotropy κ on the solution ionic strength is shown in Figure 6. The values of κ are rather small, mostly below 0.06, and noisy. Only for the lowest ionic strength is κ close to 0.1. Therefore, we did not detect any dependence of the binding anisotropy on the ionic strength. Either our mutation does not introduce a sufficient change in the electrostatic attraction/orienting of tri-*N*-acetylglucosamine toward the binding cleft or the hydrodynamic interaction does not discriminate between ligand molecules approaching the cleft with different orientations. Because our laboratory is not specialized in the expression of mutated proteins, further experiments would be rather difficult to proceed. Moreover, it is also possible that the experimental approach requires investigations of systems other than protein receptors with elongated ligands. Instead, we performed Brownian dynamics simulations of the binding process.

### Results of Brownian Dynamics Simulations

We performed Brownian dynamics simulations for an artificial model of the receptor with elongated binding cleft and elongated ligand molecule, shown in Figure 2. Brownian dynamics simulations allow us to compare the ionic strength dependence of the binding anisotropy obtained with receptor–ligand hydrodynamic interactions included and omitted during simulations.

**Figure 7:**
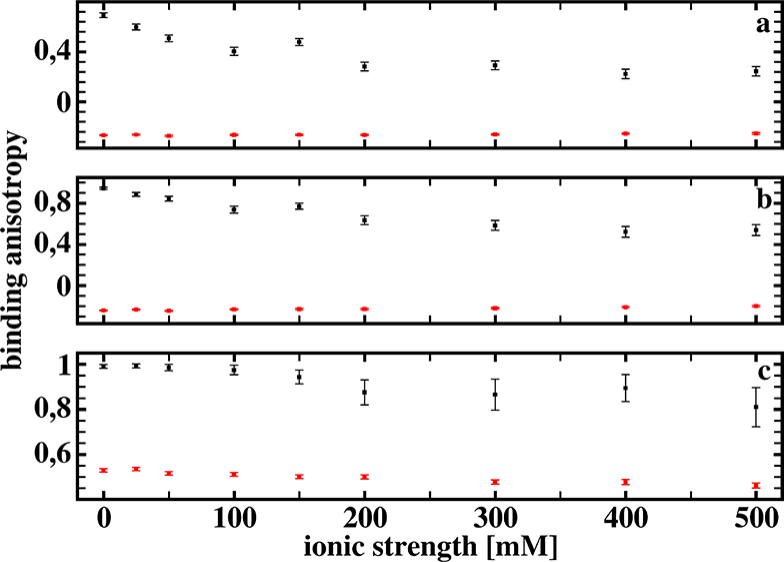
Binding anisotropy κ defined by eq 2 for the three reaction criteria, obtained from Brownian dynamics simulations with (black squares) and without (red squares) hydrodynamic interactions included, as functions of the ionic strength. See text for details.

Figure 7 presents binding anisotropy κ defined by eq 2 for the three reaction criteria, defined as described above (see Figure 3), obtained from our Brownian dynamics simulations.

As can be seen, for simulations with the hydrodynamic interactions included, all three reaction criteria result in positive values of the binding anisotropy and its increase on going from the least restrictive (a) to the most restrictive (c) criteria. For simulations without the hydrodynamic interactions included, the two less restrictive criteria give negative values of the binding anisotropy parameter κ. Thus, the number of detected reactions is larger for the receptor model with its dipole moment perpendicular to its long axis. Apparently, the electrostatic attraction of the ligand toward the area between the beads, modeling the binding site, is more effective for the mutated model than for the wild-type model of the receptor. This results in a larger number of trajectories with the ligand diffusing in the vicinity of the binding site, and the two less restrictive reaction criteria allow to classify a substantial fraction of receptor–ligand arrangements as the complex formation. Only for the most restrictive reaction criterion is the value of binding anisotropy positive; thus, the fraction of the number of receptor–ligand arrangements, classified as binding, for the model of mutated receptor must be significantly reduced. This results in positive binding anisotropies in the absence of hydrodynamic interactions.

We may conclude, based on the results shown in Figure 7, that our simple receptor–ligand model exhibits significant increase of the binding anisotropy due to hydrodynamic orienting effects. However, its ionic strength dependence does not present any particular features that would allow us to detect existence of the hydrodynamic orienting effects from this dependence alone. Having, for example, from stopped-flow experiments two ionic strength dependencies of the binding anisotropy, as those shown in the bottom part of Figure 7, we are not able to conclude that one of them has nothing to do with the hydrodynamic orienting effects. Trying to solve this problem, we determined the ionic strength dependence of the binding anisotropy for another value of the solvent viscosity. We performed an additional set of Brownian dynamics simulations for solvent viscosity 1.15 cP. This value corresponds to a 5% aqueous solution of glycerol at 293 K.^64^ The number of trajectories was again 600000 for each ionic strength.

**Figure 8:**
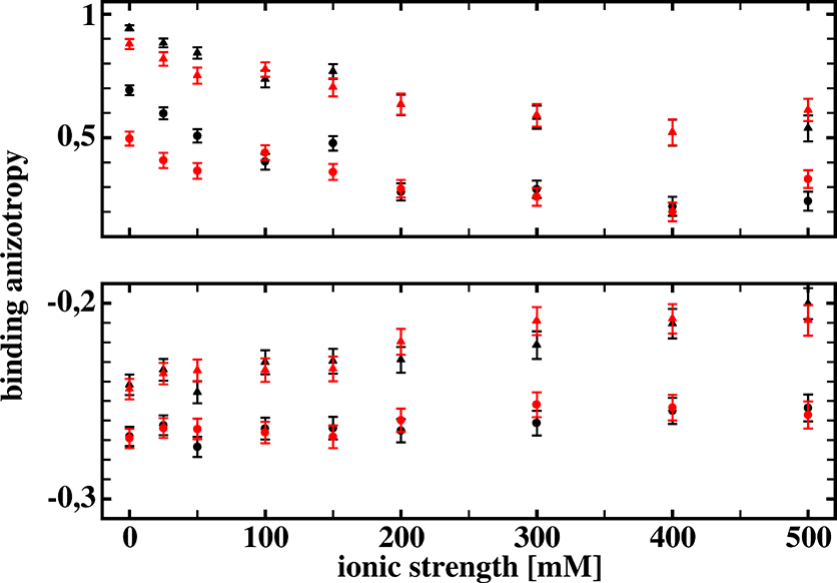
Comparison of the ionic strength dependencies of the binding anisotropy κ defined by eq 2 for the two less restrictive reaction criteria, obtained from Brownian dynamics simulations for two different solvent viscosities (1.002 cP, black; 1.150 cP, red), with (upper part) and without (bottom part) hydrodynamic interactions included. See text for details.

Figure 8 shows ionic strength dependence of the binding anisotropy κ obtained from Brownian dynamics simulations for the two values of solvent viscosity. We present here results obtained for the two less-restrictive reaction criteria. For ionic strengths below 100 mM, simulations with receptor–ligand hydrodynamic interactions included giving smaller binding anisotropies for higher viscosity. For ionic strengths above 100 mM, binding anisotropy does not exhibit viscosity dependence. There are some fluctuations seen above 100 mM, which result from relatively small number of reactions detected by our simulations. This result is qualitatively compatible with the ionic strength dependence of the relative increase in the diffusional rate constant caused by hydrodynamic torques described previously in a paper coauthored by one of the present authors (JMA^29^). In that work, this relative increase in the diffusional encounter rate constant was shown to rise in the ionic strength range from 0 to about 150 mM, reaching a plateau for the higher values.

On the other hand, for simulations without receptor–ligand hydrodynamic interactions included, there is no viscosity dependence in the whole investigated ionic strength range. Analogous results for the most restrictive reaction criterion are shown in Figure S1. We show these results in the Supporting Information because the numbers of detected reactions from simulations with the receptor–ligand hydrodynamic interactions included are relatively small. This leads to substantial fluctuations in the ionic strength dependence of the binding anisotropy for the most restrictive reaction criterion. For simulations without hydrodynamic interactions included, no viscosity dependence is visible in the whole ionic strength range.

For completeness, Tables S1–S6 present computed diffusional encounter rate constants for simulations with and without receptor–ligand hydrodynamic interactions taken into account, for the wild-type and the mutated receptor model, for all reaction criteria. As can be seen, the rate constants with hydrodynamic interactions neglected are much larger than those obtained from simulations with HI included, particularly for the model of mutated receptor. Simultaneously, it is clear from the data presented in Tables S1–S3 that the smaller values of the diffusional encounter rate constants computed for the model of mutated receptor, for all three reaction criteria, result from strong orientational steering by hydrodynamic interactions. These effects overcome stronger electrostatic attraction of the ligand by the mutated receptor visible from the simulations without hydrodynamic interactions included with the less restrictive reaction criteria. Smaller values of the rate constants for the model of mutated receptor in comparison to the wild-type receptor, obtained from Brownian dynamics simulations without hydrodynamic interactions included for the most restrictive reaction criterion, apparently result from excluded volume effects.

It might be surprising that we observe in our Brownian dynamics simulations clear effect of orientational steering by hydrodynamic interactions, whereas such effects were not visible in another Brownian dynamics simulations for the similar receptor–ligand model.^32^ Besides the differences in the sophistication of the employed models for hydrodynamic effects calculations, our being much more simplified, the simulation described in that work consisted of one-dimensional translation motion and one-dimensional rotation motion of the ligand with respect to the receptor model, whereas here the ligand has full translational and rotational freedom. The other difference is that our receptor model consists of two arrays built from five beads each, whereas in the other work the receptor model consisted of only two spherical elements. A clarification of these differences requires further investigations.

## Conclusions

The results of our Brownian dynamics simulations show that it is possible to construct simplified models of receptor–ligand exhibiting strong hydrodynamic orienting effects when, in the simulation, the ligand is approaching the binding site of the receptor. So the search for more realistic models, which will exhibit hydrodynamic orientation steering, is not doomed to failure.

On the other hand, the results of the experimental part of the project show that the association of the elongated ligand with the elongated binding cleft, as in the case of lysozyme and tri-*N*-acetylglucosamine, is not a promising receptor–ligand system for detection hydrodynamic orienting effects accompanying the formation of the encounter complex. Actually, very small values of the binding anisotropies, obtained in our experiments, strongly indicate that in the case of association of chitotriose with elongated binding cleft of lysozyme hydrodynamic orienting interactions apparently play no role. Therefore, one has to look for more complex molecular shapes among receptor–ligand systems available for experimental kinetic investigations, which can be modeled by relatively small number of spherical elements.

The most important conclusion from the present work is the following. Assume that one finds a receptor–ligand system that exhibits in experiments substantial binding anisotropy. Moreover, the accuracy of the measurements is high. Then, one needs to repeat the experiments in a solvent of different viscosity. That allows one to see if the anisotropy has only electrostatic origins. The viscosity dependence of the anisotropy might indicate hydrodynamic orienting contributions.

## Supporting Information Available

Figure S1, Tables S1–S6, comments to data presented in these tables, and examples of input files for the UHBD program, for simulations with and without receptor–ligand hydrodynamic interactions included (PDF)
