# Supplementary material for: Searching for Hydrodynamic Orienting Effects in the Association of Tri-N-acetylglucosamine with Hen Egg-White Lysozyme
Source: J Phys Chem B. 2021 Sep 21;125(38):10701–9. doi: 10.1021/acs.jpcb.1c06762 (PMC8488934; doi:10.1021/acs.jpcb.1c06762)
Supplement: Supplementary file 1 — jp1c06762_si_001.pdf [file jp1c06762_si_001.pdf]

Supporting information for: Searching for Hydrodynamic Orienting Effects in the Association of Tri-N-acetylglucosamine with Hen Egg-White Lysozyme

Beata Wielgus-Kutrowska, Urszula Marcisz, and Jan M. Antosiewicz*

Biophysics Division, Institute of Experimental Physics, Faculty of Physics, University of Warsaw, Pasteura 5 St., 02-093 Warsaw, Poland

E-mail: jantosi@fuw.edu.pl

Phone: +48 22 55 32 340

This file includes Supporting Figure S1, Supporting Tables S1 to S6, comments to the data presented in these Tables, and examples of the UHBD program inputs.

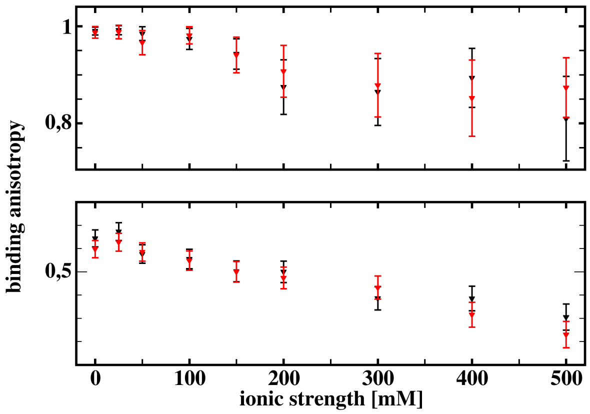

Figure S1: Comparison of the ionic strength dependencies of the binding anisotropy κ defined by Equation 2, for the most restrictive reaction criterion, obtained from Brownian dynamics simulations for two different solvent viscosities (1.002 cP black, 1.150 cP red), with (upper part) and without (bottom part) hydrodynamic interactions included. See text for details.

.

Table S1: Diffusional encounter rate constants, obtained from Brownian dynamics simulations, and their standard errors, obtained for the reaction criterion 1, for 'the "wild type" receptor model, k_a^{WT}, and the mutated receptor model, k_a^{MT}, with hydrodynamic interactions included during simulations. See text for details.

Ionic Strength [mM]	viscosity [cP]	k_a^{WT} [μM^{-1}s^{-1}]	StdErr [μM^{-1}s^{-1}]	k_a^{MT} [μM^{-1}s^{-1}]	StdErr [μM^{-1}s^{-1}]
0.	1.002	415	21	75.5	4.3
	1.150	165	9	55.5	3.1
25.	1.002	299	15	75.3	4.3
	1.150	126	7	53.1	2.9
50.	1.002	245	12	79.9	4.4
	1.150	119	6	55.3	3.1
100.	1.002	181	9	77.1	4.3
	1.150	133	7	51.9	2.9
150.	1.002	216	11	76.4	4.4
	1.150	118	6	55.3	3.1
200.	1.002	146	8	81.8	4.6
	1.150	92.9	4.8	50.7	3.0
300.	1.002	167	9	92.2	5.5
	1.150	83.1	4.4	48.7	2.9
400.	1.002	137	8	87.3	
	1.150	70.3	3.7	46.8	2.8
500.	1.002	119	7	72.6	4.5
	1.150	90.2	4.9	45.1	2.8

Table S2: Diffusional encounter rate constants, obtained from Brownian dynamics simulations, and their standard errors, obtained for the reaction criterion 2, for 'the "wild type" receptor model, k_a^{WT}, and the mutated receptor model, k_a^{MT}, with hydrodynamic interactions included during simulations. See text for details.

Ionic Strength [mM]	viscosity [cP]	k_a^{WT} [μM^{-1}s^{-1}]	StdErr [μM^{-1}s^{-1}]	k_a^{MT} [μM^{-1}s^{-1}]	StdErr [μM^{-1}s^{-1}]
0.	1.002	239	16	6.83	1.29
	1.150	95.2	6.3	6.13	1.02
25.	1.002	168	12	10.4	1.6
	1.150	72.3	4.8	7.21	1.08
50.	1.002	136	10	11.6	1.7
	1.150	65.1	4.5	9.25	1.24
100.	1.002	95.9	6.8	14.5	1.9
	1.150	72.9	5.0	9.20	1.23
150.	1.002	115	8	15.1	2.0
	1.150	60.4	4.3	10.5	1.4
200.	1.002	77.5	5.5	17.4	2.1
	1.150	48.3	3.5	10.8	1.4
300.	1.002	82.5	6.1	21.7	2.7
	1.150	43.3	3.2	11.1	1.4
400.	1.002	65.3	5.3	20.6	2.7
	1.150	34.3	2.6	10.8	1.4
500.	1.002	56.7	4.5	17.0	2.2
	1.150	45.8	3.5	11.0	1.4

Table S3: Diffusional encounter rate constants, obtained from Brownian dynamics simulations, and their standard errors, obtained for the reaction criterion 3 (the most restrictive one), for 'the "wild type" receptor model, k_a^{WT}, and the mutated receptor model, k_a^{MT}, with hydrodynamic interactions included during simulations. See text for details.

Ionic Strength [mM]	viscosity [cP]	k_a^{WT} [μM^{-1}s^{-1}]	StdErr [μM^{-1}s^{-1}]	k_a^{MT} [μM^{-1}s^{-1}]	StdErr [μM^{-1}s^{-1}]
0.	1.002	71.7	8.6	0.355	0.292
	1.150	30.0	3.6	0.190	0.181
25.	1.002	44.2	5.8	0.177	0.206
	1.150	20.0	2.5	0.120	0.140
50.	1.002	34.9	4.7	0.266	0.253
	1.150	17.7	2.5	0.304	0.224
100.	1.002	27.0	3.6	0.356	0.293
	1.150	19.1	2.6	0.181	0.172
150.	1.002	28.1	4.0	0.824	0.452
	1.150	14.2	2.1	0.434	0.270
200.	1.002	18.3	2.7	1.22	0.56
	1.150	10.4	1.6	0.505	0.294
300.	1.002	16.5	2.8	1.20	0.63
	1.150	8.49	1.39	0.548	0.301
400.	1.002	16.2	2.6	0.905	0.527
	1.150	6.83	1.16	0.545	0.300
500.	1.002	11.5	2.0	1.20	0.57
	1.150	10.1	1.7	0.677	0.337

Table S4: Diffusional encounter rate constants, obtained from Brownian dynamics simulations, and their standard errors, obtained for the reaction criterion 1, for 'the "wild type" receptor model, k_a^{WT}, and the mutated receptor model, k_a^{MT}, with hydrodynamic interactions not included during simulations. See text for details.

Ionic Strength [mM]	viscosity [cP]	k_a^{WT} [$\mu M^{-1}s^{-1}$]	StdErr [$\mu M^{-1}s^{-1}$]	k_a^{MT} [$\mu M^{-1}s^{-1}$]	StdErr [$\mu M^{-1}s^{-1}$]
0.	1.002	4130	36	7150	44
	1.150	2750	24	4780	29
25.	1.002	3900	35	6670	43
	1.150	2600	24	4470	28
50.	1.002	3670	34	6440	42
	1.150	2470	23	4250	28
100.	1.002	3410	33	5860	41
	1.150	2290	22	3950	27
150.	1.002	3180	32	5470	40
	1.150	2120	22	3670	26
200.	1.002	3030	32	5220	39
	1.150	2020	21	3430	26
300.	1.002	2760	30	4710	37
	1.150	1860	20	3120	25
400.	1.002	2580	30	4340	36
	1.150	1710	20	2870	25
500.	1.002	2400	29	4030	35
	1.150	1600	19	2710	24

Table S5: Diffusional encounter rate constants, obtained from Brownian dynamics simulations, and their standard errors, obtained for the reaction criterion 2, for 'the "wild type" receptor model, k_a^{WT}, and the mutated receptor model, k_a^{MT}, with hydrodynamic interactions not included during simulations. See text for details.

Ionic Strength [mM]	viscosity [cP]	k_a^{WT} [μM^{-1}s^{-1}]	StdErr [μM^{-1}s^{-1}]	k_a^{MT} [μM^{-1}s^{-1}]	StdErr [μM^{-1}s^{-1}]
0.	1.002	3860	36	6324	42
	1.150	2580	23	4250	28
25.	1.002	3620	34	5830	41
	1.150	2420	23	3920	27
50.	1.002	3400	33	5610	40
	1.150	2290	22	3690	26
100.	1.002	3110	32	4980	38
	1.150	2090	21	3370	26
150.	1.002	2860	31	4570	37
	1.150	1910	20	3080	25
200.	1.002	2700	30	4310	36
	1.150	1810	20	2820	24
300.	1.002	2420	29	3790	34
	1.150	1630	19	2500	23
400.	1.002	2220	28	3400	33
	1.150	1470	18	2250	22
500.	1.002	2050	26	3070	32
	1.150	1360	18	2080	22

Table S6: Diffusional encounter rate constants, obtained from Brownian dynamics simulations, and their standard errors, obtained for the reaction criterion 3 (the most restrictive one), for 'the "wild type" receptor model, k_a^{WT}, and the mutated receptor model, k_a^{MT}, with hydrodynamic interactions not included during simulations. See text for details.

Ionic Strength [mM]	viscosity [cP]	k_a^{WT} [$\mu M^{-1}s^{-1}$]	StdErr [$\mu M^{-1}s^{-1}$]	k_a^{MT} [$\mu M^{-1}s^{-1}$]	StdErr [$\mu M^{-1}s^{-1}$]
0.	1.002	3580	34	1100	20
	1.150	2390	22	755	13
25.	1.002	3320	33	1010	19
	1.150	2120	22	689	13
50.	1.002	3070	32	983	19
	1.150	2080	21	661	12
100.	1.002	2760	30	894	18
	1.150	1870	20	607	12
150.	1.002	2510	29	836	17
	1.150	1680	19	558	12
200.	1.002	2350	28	782	17
	1.150	1570	19	529	11
300.	1.002	2030	26	718	16
	1.150	1370	18	474	11
400.	1.002	1820	25	644	15
	1.150	1210	17	444	10
500.	1.002	1630	24	603	15
	1.150	1090	16	416	10

.

.

Comments to data presented in Tables S1 to S6

At first glance, the differences between rate constants computed with hydrodynamic interactions included (Tables S1–S3) and neglected (Tables S4–S6), respectively, may seem much too large. Most of the previous studies reported a substantially smaller decrease in the association rate constants caused by the inclusion of HI between two spherical particles[S1,S2] or a spherical target and dumbbell dimer.[S3] In these studies, spherical elements used to model molecules were considered uniformly reactive over their surfaces, and a single distance criterion was used to define association reactions. On the other hand, Shushin[S4] analyzed the effect of hydrodynamic interaction on diffusion-controlled reaction rate of molecules with highly anisotropic reactivity, modeled by small reactive hemispheres around the reactive centers on the surfaces of spherical molecules. He showed that the hydrodynamic interaction effect can lead to about 3-5 times and larger reduction of the rate. In the case of our simulations, we obtain such reductions as 50 (ionic strength 0) and 108 (ionic strength 500 mM) for the most restrictive reaction criterion, and as 10 (ionic strength 0) and 18 (ionic strength 500 mM) for the least restrictive reaction criterion. The increase in the ratio with going from the least to the most restrictive reaction criterion is in qualitative agreement with the results of Shushin. We believe that more complex molecular shapes and requirement to satisfy simultaneously four distance criteria instead of just one are responsible for the higher values in our case.

Finally, in a recent publication on encounter rates between xanthone and 2-naphtoic acid,[S5] we also obtained substantial decrease of the association rate with hydrodynamic interactions included in comparison to hydrodynamic interactione neglected, i.e 10250/723=14. In this case we deal with molecular models composed of several spherical elements, but the reaction criteria used a single distance. It is worthy to note that the rate constant obtained for simulations neglecting receptor-ligand hydrodynamic interactions, 10250 $\mu M^{-1}s^{-1}$, for

xanthone and 2-naphthoic acid was shown to be in excellent agreement with the result of the analytical equation of Smoluchowsky, 11146 $\mu M^{-1} s^{-1}$.

It may be also noted that that the decrease of the encounter rates with increased solvent viscosity is somewhat larger than predicted by the analytical Smoluchowsky equation for spherical particles, as according to this equation the association rate constant is proportional to the inverse of viscosity of the solvent. However, it should be also noted that no-HI simulation means that there is no hydrodynamic interactions between beads of the receptor model with the beads of the ligand model. The receptor is at rest in the center of the coordinate system, with its hydrodynamic radius of equivalent spere computed assuming hydrodynamic interactions between constituting beads. On the other hand, the ligand diffusion is simulated with hydrodynamic interactions between its constituting beads included. Moreover, it is probably also important that four distances are used in definition of the reaction criteria instead of just one. Thus simple recalculation referring to association of single spheres may be misleading.

Examples of the UHBD program inputs

Simulations with receptor-ligand hydrodynamic interactions included

```
read mol1 file "./enzym-10bead-model.pdb" pdb end ! read in receptor
read mol2 file "./ligand-3bead-model.pdb" pdb end ! read in ligand
set charge radii file "./qrdata.dat"              ! set charges and ra
    para par end                                  ! using data in file
                                                  ! qrdata.dat

read phi grid file "enzyme-par.pot" binary end
elec setup same grid                      ! no electrostatic calcul
  nsph 280
  sdie 78.0                               ! solvent dielectric = 78
  pdie 4.0                                ! protein dielectric =  4
  temp 293.0
  ionstr 0.0
  nmap 1.4
end

edit suni iflx 1
    numsub 10 atnum 1 1 2 2  3 3  4 4  5 5  6 6 7 7  8 8  9 9  10 10
    cent atnum 1 2 3 4 5 6 7 8 9 10
    fix 4 atnum 1 5 6 10
    prts
end

edit suni iflx 2
     numsub 3 atnum 11 11  12 12  13 13
```

```
     cent atnum 11  12  13
     fix 0
     prts
end

edit funi iflx 1
        oldb
loccor dfcnsr 10
1  2 3.00
2  3 3.00
3  4 3.00
        4  5 3.00
        6  7 3.00
        7  8 3.00
        8  9 3.00
        9 10 3.00
1  6 12.00
        5 10 12.00
        toler 0.10
mdoseen stick hrident 2.00
hi 15
   2 3
   2 4
   2 7
   2 8
   2 9
   3 4
```

```
   3 7
   3 8
   3 9
   4 7
   4 8
   4 9
   7 8
   7 9
   8 9
nbon
nexv
nang
nsol
nonnnb
prts
prin
end


edit funi iflx 2
     oldb
     loccor dfcnsr 3
     11 13 6.000
     11 12 3.000
     12 13 3.000
     toler 0.10
     mdoseen stick hrident 2.00
```

```
     hi 3
        11 12
        11 13
        12 13
nbon
nexv
nang
nsol
nonnnb
prts
prin
end

edit fiun
    num 3  0 1 mdoseen stick nexv nsol end
           0 2 mdoseen stick nexv nsol end
           1 2 mdoseen stick end
    prnt
end

bd calc nruns 1 ntraj 3000  ! 1 run of bd, 1000 traj
   srad 2.0                 ! effective substrate excl
                            !  radius
   rad1 7.83                ! hydrodynamic radius of targ
   dfcr 100                 ! tries to estimate relati
                            !  diffusion coefficent
```

```
temp 293.0

svis 1.002


psurf 30.0

bsurf  55.0

qsurf 110.0


delt 0.010

vtim 4 30.0 2.0 45.0 5.0 50.0 10.0 60.0 20.0


     ijsd1    10710

     klsd1    11800

     ijsd2     9301

     klsd2    16640


mrxn defrxn  3 4 2 11  9.0

                 7 11  9.0

                 4 13  9.0

                 9 13  9.0

               4 2 11  8.0

                 7 11  8.0

                 4 13  8.0

                 9 13  8.0

               4 2 11  7.0

                 7 11  7.0

                 4 13  7.0

                 9 13  7.0
```

```
     trjst
end
stop
```

Simulations with receptor-ligand hydrodynamic interactions neglected

```
read mol1 file "./enzym-10bead-model.pdb" pdb end  ! read in receptor
read mol2 file "./ligand-3bead-model.pdb" pdb end  ! read in ligand
set charge radii file "./qrdata.dat"               ! set charges and ra
    para par end                                   ! using data in file
                                                   ! qrdata.dat

read phi grid file "enzyme-par.pot" binary end
elec setup same grid                           ! no electrostatic calcul
  nsph 280
  sdie 78.0                                    ! solvent dielectric = 78
  pdie 4.0                                     ! protein dielectric =  4
  temp 293.0
  ionstr 0.0
  nmap 1.4
end

edit suni iflx 1
     numsub 3 atnum 11 11  12 12  13 13
         cent atnum 11  12  13
     fix 0
     prts
end
```

```
edit funi iflx 1
     oldb
     loccor dfcnsr 3
     1 3 6.000
     1 2 3.000
     2 3 3.000
     toler 0.10
     mdoseen stick hrident 2.00
     hi 3
        1 2
        1 3
        2 3
nbon
nexv
nang
nsol
nonnnb
prts
prin
end

bd calc nruns 1 ntraj 3000      ! 1 run of bd, 1000 traj
   srad 2.0                     ! effective substrate excl
                                !  radius
   rad1 7.83                    ! hydrodynamic radius of targ
   dfcr 100                     ! tries to estimate relati
                                !  diffusion coefficent
```

```
temp 293.0
svis 1.002

psurf 30.0
bsurf  55.0
qsurf 110.0

delt 0.010
vtim 4 30.0 2.0 45.0 5.0 50.0 10.0 60.0 20.0

     ijsd1     9073
     klsd1    30031
     ijsd2     4636
     klsd2    21612

mrxn defrxn  3 4 2 1  9.0
                   7 1  9.0
                   4 3  9.0
                   9 3  9.0
                 4 2 1  8.0
                   7 1  8.0
                   4 3  8.0
                   9 3  8.0
                 4 2 1  7.0
                   7 1  7.0
                   4 3  7.0
```

```
                    9 3  7.0
     trjst
end
stop
```

References

(S1) Friedman, H. L. A Hydrodynamic Effect in the Rates of Diffusion Controlled Reactions. *J. Phys. Chem.* **1966**, *70*, 3931–3933.

(S2) Deutch, J. M.; Felderhof, B. U. Hydrodynamic Effect in Diffusion-Controlled Reaction. *J. Chem. Phys.* **1973**, *59*, 1669–1671.

(S3) Allison, S. A.; Srinivasan, N.; McCammon, J. A.; Northrup, S. H. Diffusion-Controlled Reactions Between a Spherical Target and Dumbell Dimer by Brownian dynamics Simulation. *J. Phys. Chem.* **1984**, *88*, 6152–6157.

(S4) Shushin, A. I. Influence of Hydrodynamic Interaction on the Diffusion-Controlled Reaction Kinetics of Molecules with Highly Anisotropic Reactivity. *J. Chem. Phys.* **2003**, *118*, 1301–1311.

(S5) Stachurska, K.; Grochowski, P.; Antosiewicz, J. M. Diffusional Encounter Rate Constants for Xanthone and 2-Naphthoic Acid by Flash Photolysis Experiments and Brownian Dynamics Simulations: Substantial Effects of Polarizability of the Triplet State. *J. Phys. Chem. B* **2019**, *123*, 9328–9342.
